# A national virtual education program to promote the brain health of aging adults with intellectual and developmental disabilities

**DOI:** 10.1002/alz70858_104111

**Published:** 2025-12-26

**Authors:** Anupam Thakur, Nicole Bobbette, Mary Chiu, Prachi Patel, Tiziana Volpe, Yona Lunsky

**Affiliations:** ^1^ CAMH, Toronto, ON, Canada; ^2^ Queen's University, Kingston, ON, Canada; ^3^ Ontario Shores Centre for Mental Health Sciences, Whitby, ON, Canada; ^4^ Centre for Addiction and Mental Health, Toronto, ON, Canada

## Abstract

**Background:**

Adults with intellectual and developmental disabilities (IDD) are living longer due to improvements in health and social care. However, aging adults with IDD face an increased risk of dementia, mood and anxiety. As this population grows, it is crucial to build clinical capacity to support their unmet physical and mental health needs. Project Extension of Community Health Outcomes, a virtual telementoring program, has proven effective in training service providers supporting adults with IDD. This study describes the implementation and evaluation of a national virtual education program focused promoting brain health for aging adults with IDD.

**Methods:**

The national Brain Health – Intellectual and Developmental Disabilities (BH‐IDD) program consists of six weekly sessions, each lasting 1.5‐hours. Each session includes didactic teaching and a 30‐45 minute case‐based discussion. Session topics include an overview of brain health, physical and mental health issues in aging, dementia screening and care, navigating change, and building resilience. Along with health care experts, people with lived experience (adults with IDD and family caregivers) were involved in the co‐design and delivery of the program. Moore's evaluation framework was used, and focused on participation, satisfaction, learning, self‐efficacy, and change in practice. Participants rated these domains on a 5‐point scale and qualitative feedback from open‐text responses were also analyzed.

**Results:**

A total of 140 care providers from health and disability service sectors participated in the first two cycles. High levels of engagement (105 attended three or more sessions) and satisfaction (overall satisfaction score: mean 4.35, SD 0.10) were observed. Self‐efficacy ratings improved from pre (64.54 ± 22.48) to post‐program (78.63 ± 16.45) at a significant level (*p* < 0.0001). The majority of participants agreed that the involvement of adults with IDD (92.10%) and family members (89.92%) enhanced their learning. Participants also reported that the inter‐professional aspect of the program enriched their learning.

**Discussion:**

The BH‐IDD program is an effective capacity‐building model with a shared‐learning approach. This study also shows the valuable role of people with lived experience in fostering learning to promote brain health. Future studies should explore the educational impact of such programs on care delivery and health outcomes.

